# Validation of the FRESH Austin food frequency questionnaire using multiple 24-h dietary recalls

**DOI:** 10.1017/S1368980021002214

**Published:** 2022-06

**Authors:** Christine ES Jovanovic, Jacob Whitefield, Deanna M Hoelscher, Boajiang Chen, Nalini Ranjit, Alexandra E van den Berg

**Affiliations:** 1 University of Texas, School of Public Health, 1705 Guadalupe St, 2nd Floor, Austin, TX 78701, USA; 2 Texas Health and Human Services Commission, Austin, TX, USA; 3 University of Texas, School of Public Health, Austin, TX, USA

**Keywords:** Validation, Food frequency questionnaire, 24hDR

## Abstract

**Objective:**

The purpose of the current study was to examine the validity of an FFQ utilised in the Food Retail: Evaluating Strategies for a Healthy Austin (FRESH Austin) study, designed to evaluate changes in the consumption of fruits and vegetables (FV) in diverse low-income communities in Austin, TX.

**Design:**

The FRESH Austin FFQ was validated against three 24-h dietary recalls (24hDR). All dietary assessments were administered (in-person or by telephone) by trained investigators.

**Setting:**

Recruitment was conducted at sites within the geographic areas targeted in the FRESH Austin recruitment. People at a community health clinic, a local health centre and a YMCA within the intervention area were approached by trained and certified data collectors, and invited to participate.

**Participants:**

Among fifty-six participants, 83 % were female, 46 % were non-White, 24 % had income < $25 K/year and 30 % spoke only/mostly Spanish at home.

**Results:**

The FFQ and average of three 24hDR produce similar estimates of average total servings/d across FV (6·68 and 6·40 servings/d, respectively). Correlations produced measures from 0·01 for ‘Potatoes’ and 0·59 for ‘Other Vegetables’. Mean absolute percentage errors values were small for all FV, suggesting the variance of the error estimates was also small. Bland–Altman plots indicate acceptable levels of agreement between the two methods.

**Conclusion:**

These outcomes indicate that the FRESH FFQ is a valid instrument for assessing FV consumption. The validation of the FRESH Austin FFQ provides important insights for evaluating community-based efforts to increase FV consumption in diverse populations.

Evaluating dietary outcomes of community-based interventions is challenging, requiring both measurable change at the individual level and the instruments to detect that change. Often, because effects of community-level interventions are broad and diffuse, individual-level outcomes are difficult to capture. Historically, dietary assessment generally has focused on producing precise measures of micronutrients in an effort to discover epidemiological relations with disease outcomes. More recently, however, the focus has shifted to assessing changes in habitual dietary patterns at the food group level (e.g., fruits, vegetables, meat)^(1–3)^. This reflects the intention of community-based interventions to increase/decrease consumption of specific foods or food groups (e.g., increasing consumption of fruits and vegetables (FV)), rather than targeting a change in micronutrient consumption (e.g., increasing K intake)^(4)^.

Among the variety of dietary assessment methods available, the FFQ may be the best option for community-based programme evaluation, as it captures usual intake in a cost-effective and minimally burdensome process^(5)^. Best practice recommends that FFQ be tailored to both the study aims and the study population, so that the foods queried reflect the outcomes of interest, as well as the culture and usual diet of study participants^(6)^. These adaptations may change the validity of the instrument, however, and ideally instruments should be validated when adapted to new studies and specific study populations^(7,8)^. Thus, the purpose of the current study was to examine the validity of an FFQ utilised in the Food Retail: Evaluating Strategies for a Healthy Austin (FRESH Austin) study, designed to evaluate changes in the consumption of FV in diverse low-income communities in Austin, TX. In alignment with well-established dietary assessment protocols, repeated 24-h dietary recalls (24hDR) serve as the criterion measure^(9–11)^.

## Methods

### Food Retail: Evaluating Strategies for a Healthy Austin parent study

FRESH Austin is a study that evaluates the fresh for less (FFL) initiative, which aims to improve access to healthy, affordable food in ethnically diverse and economically disadvantaged communities through organisational support of local mobile markets in Austin, TX^(12)^. By decreasing barriers to healthy food access, FFL is intended to affect purchasing behaviours and, ultimately, increase the consumption of fresh FV in the target communities. Because increased consumption of fresh FV is the primary outcome of interest, the FRESH Austin survey includes an FFQ that queried only fresh FV, rather than the entire diet. The FRESH Austin survey, which includes the FFQ, is being administered to a cohort of 400 residents over a 3-year time period. This validation study is intended to assess the validity of the FRESH Austin FFQ in comparison to three 24hDR, focusing on assessing the consumption of FV, rather than the entire diet.

### Participants

Because the purpose of the current study was to validate the FRESH Austin FFQ, participants were recruited in a similar way across both studies: by location within the priority areas identified and served by the FFL. Inclusion criteria for both FRESH Austin and the validation study were the same: at least 18 years old, not pregnant or breast-feeding and able to speak English or Spanish.

### Data collection protocol

Recruitment was conducted at sites within the geographic areas targeted in the FFL intervention, which were the same areas in Austin where the FRESH Austin study was situated. People at a community health clinic, a local health centre and a YMCA within the FFL intervention area were approached by trained and certified data collectors, and invited to participate in the validation study. As in the FRESH Austin study, all the materials (consent, information sheet, survey) for the validation study were available in English and Spanish, and trained bilingual research staff conducted the data collection in either Spanish or English, as the participant preferred. In accordance with the approved IRB protocol, participants were given an information sheet, were invited to ask questions, and, if they were willing to participate, signed an IRB-approved consent. All protocols and instruments were approved by the University of Texas Internal Review Board, HSC-SPH-18-0233.

Research shows that correlations between FFQ and reference methods such as repeated 24hDR are higher for interviewer-conducted FFQ, as compared with those that are self-administered^(13,14)^. However, no difference in correlation has been found between FFQ conducted via telephone interview with a qualified researcher and those conducted in-person^(14,15)^. Therefore, the FRESH Austin validation study protocol included either in-person or telephone interviews with trained personnel. This also aligns with the protocol used in the FRESH Austin study for data collection. Using the multiple-pass method developed and validated by the United States Department of Agriculture (USDA)^(16,17)^, three 24hDR were administered to each participant, followed by the FRESH Austin FFQ, at four separate times. At the time of recruitment, the first 24hDR was administered in-person, and arrangements were made for the second 24hDR via telephone interview. At the time of the second 24hDR, a day and time for the third 24hDR was arranged. After three 24hDR were completed, the FRESH Austin FFQ was conducted either in-person or over the phone, depending on the availability and preference of the participant, and incentives were delivered. For both FRESH Austin and this validation study FFQ, all reported food amounts were described in terms of cups, and these amounts were compared with a hand graphic measurement guide, which featured comparisons such as a fist to a one cup portion of vegetables^(18)^. This allowed for a simple and consistent method of estimating portion sizes across both the FFQ and the 24hDR.

In all, sixty-nine people were recruited; five chose not to continue after the first 24hDR interview, six declined after the second and four chose not to complete the final FFQ, resulting in a final sample size of fifty-four. Participants were classified as dropped from the study after four attempts to reach the participant were made, or the participant requested to leave the study. Participants received a total of $20 in gift cards for completing all four assessments.

### Food Retail: Evaluating Strategies for a Healthy Austin validation study survey

The FFQ in the FRESH Austin survey was adapted from the Block FFQ, a semi-quantitative assessment tool which is used in the NHANES annual survey and has been widely validated^(19–21)^. The foods in the FRESH Austin FFQ were chosen to capture the most commonly consumed FV in the study population (diverse, low-income population in Austin, TX)^(22)^, taking into account local sales data^(23)^ as well as feedback from *promotoras* who work in the study communities. The question stems were, ‘Over the last month, how many times/month, week, or day did you eat the following fruit/vegetable?’ and, ‘When you ate the fruit/vegetable, how much did you usually eat?’ The FV listed were apples, citrus, bananas, berries, grapes, melon, lettuce, dark leafy greens, broccoli or cauliflower, carrots, tomatoes, avocadoes, sweet potatoes, potatoes (not sweet), cabbage, peppers, maize, zucchini or other squash, and onions. In addition, respondents were given an option to mention up to four additional fruits and four additional vegetables not included in this listing.

**Fig. 1:**
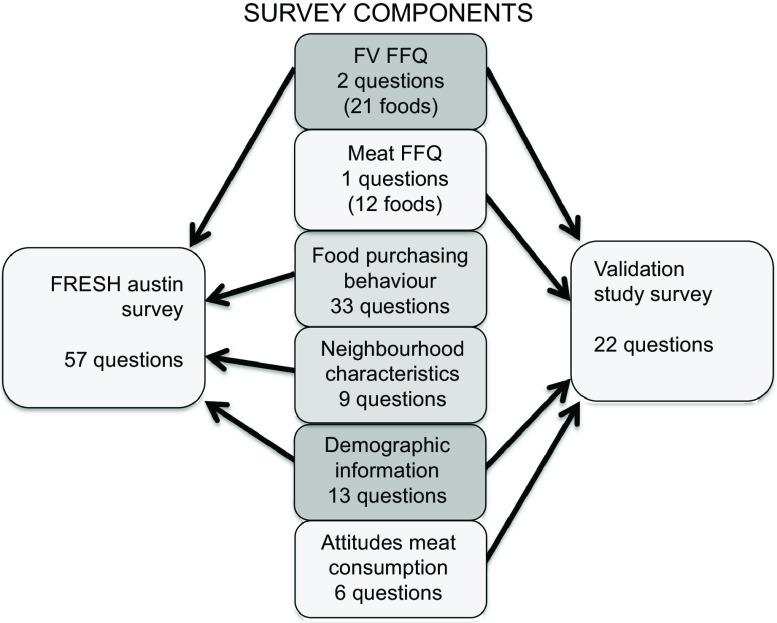
Components of the Food Retail: Evaluating Strategies for a Healthy (FRESH) Austin survey and the FRESH Austin validation study survey

This validation study examines only the FRESH Austin FFQ (Fig. 1), along with pertinent demographic questions. In all, the FRESH Austin validation study survey contained twenty-two questions and was administered in either Spanish or English, as preferred by the participant.

### Twenty-four hour dietary recalls

The USDA five-step multiple-pass 24hDR method has been shown to capture dietary energy and macronutrient intake within 10 % of actual intake, as determined by estimated energy requirements and BMR^(24)^. This allows the 24hDR to be used as the ‘gold standard’ against which the accuracy of the FFQ can be measured^(16,17)^. Our study utilised three 24hDR, conducted using the five-step multiple-pass method, and was guided by scripts adapted from those provided by Nutrition Data Systems for Research (NDSR). To ensure that the data covered the same time period defined in the FRESH Austin FFQ (i.e., the 30 d), three recalls were completed in a period of 30 d, with two recalls of weekdays and one of a weekend day. A visual hand guide to portion sizes^(18)^ was employed in all data collections (both FRESH Austin and the validation study) to assist participants in reporting portion sizes. Dietary intake data were entered and analysed using NDSR-2018 software (NDSR version 2008; Nutrition Coordinating Center, University of Minnesota)^(25)^. The NDSR utilises the USDA food composition database, which is maintained by the Agricultural Research Service^(26)^.

### Data preparation

To minimise discrepancies in data entry, all 24hDR records were entered by two trained personnel (M.D. and J.W.), and then crosschecked in their entirety, and disagreements resolved collaboratively. In consultation with the FRESH Austin team, which includes experts in nutritional sciences, final categories of FV were chosen based on congruence between the FFQ and 24hDR. For example, the ‘Deep Yellow’ vegetable category from the 24hDR was mapped to sweet potatoes and carrots from the FFQ, while the ‘Dark Green’ category from the 24hDR was mapped to cooked dark leafy greens from the FFQ (Table 1). Final categories for fruit were citrus and non-citrus fruit, and final categories of vegetables were avocados, dark green, deep yellow, tomatoes, white potatoes, starchy and other vegetables. All quantities reported are expressed in servings/d, and all data are used, so that if a fruit or vegetable did not fall into one of the defined categories, it was included in ‘Other’. In accordance with USDA convention, servings are defined as: half a cup of any cooked or raw vegetable or fruit or one cup of raw leafy greens. For a person on a 8368 kJ/d (2000 kcal/d) diet, the Recommended Dietary Guidelines for Americans 2015–2020 suggest 2·5 cups of vegetables and 2 cups of fruit/d, or 9–10 servings total^(27)^. Servings per day for each FV were generated by converting weekly or monthly consumption to daily, and multiplying this by servings as defined above. All variables are continuous measures of servings/d. Data were examined for outliers. Any values +/- two standard deviations were re-examined for plausibility and data collectors queried to confirm accuracy of all extreme values.

**Table 1:** Validation study of food categories and their components from FRESH Austin FFQ and repeated 24hDR data

Food category	FFQ item(s)	24hDR item(s)*
Citrus	Citrus (oranges, grapefruit, etc.)	Citrus fruit (oranges, grapefruit, tangerines, lemons)
Non-citrus fruits	Apples, bananas, berries, grapes, melon other non-citrus	Fruit excluding citrus fruit
Avocados	Avocados	Avocados
Dark green	Lettuce or raw dark leafy greens, cooked dark leafy greens, broccoli or cauliflower	Spinach, collards, romaine, broccoli
Deep yellow	Carrots, sweet potatoes	Carrots, sweet potatoes, pumpkin, winter squash
Tomatoes	Tomatoes	Tomatoes
White potatoes	Potatoes (not sweet)	White potatoes, fried potatoes
Starchy	Maize, peas, jicama	Peas, maize, cassava, jicama
Other vegetables	Other	Other

24hDR, 24-h dietary recalls; NDSR, Nutrition Data System for Research.

* Categories based on NDSR classification.

### Statistical analysis

Demographic characteristics of participants in the validation study and the FRESH Austin study were compared using *χ* ^2^ tests for sex, ethnicity, income and language at home (frequency and percentage reported) or *t*-test for age (mean and sd reported). In addition, scatterplots comparing categories of FV for the FFQ *v*. the 24hDR were generated for each of the nine food categories and examined for linearity (not provided). Crude mean and sd for categories of FV, separately and together, were computed. Shapiro–Wilks tests assessed normality of each food category variable. For non-normal variables, Spearman’s *ρ* was reported, and Pearson’s *r* was reported for normally distributed variables. Because the paired *t*-test is robust to non-normal distributions in larger (*n* > 30) samples, this was used to assess differences between mean values for each food category, and *P*-values were reported^(28)^. Correlation estimates (Pearson’s *r* or Spearman’s *ρ*) provide a measure of the relationship between the FFQ and the 24hDR. In order to adjust for random error from within-subjects variation across repeated 24hDR, correlations were deattenuated using the formula:

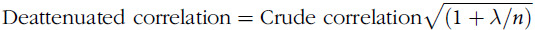




where *λ* is the ratio of within- to between-subject variance and *n* is the number of replicates of dietary data^(29)^. Within- and between-subject variances were obtained using one-way ANOVA of the 24hDR.

Correlations can be misleading if they are caused by a widespread sample (as when disagreement between methods is large but linear) and only provide an indication of the strength of the linear relationship between variables, rather than defining agreement. Therefore, we also present Bland–Altman plots, which plot the difference of paired variables *v*. their average, allowing estimates of fixed bias via mean difference. This bias is deemed significant based on its variance from zero. Limits of agreement (i.e., mean difference ± 1·96 sd of the difference) provide an estimate of variability of the agreement between the two methods and describe the range of values in which agreement between methods will fall for 95 % of the sample^(30)^. Bland–Altman plots were also used to explore proportional bias, which occurs when differences between methods vary across the sample.

Finally, borrowing from methods used in time series forecast analyses, mean absolute percentage errors (MAPE) provide a measure of percentage error between the paired FFQ and 24hDR observations, which can be indicative of prediction accuracy. These estimates use the 24hDR as the criterion or actual value and the FFQ as the forecast or predicted value, scaled to each category. Lower estimates suggest lower error, and better prediction, of the FFQ from the 24hDR. Significance was set at *α* = 0·05 for all tests, and all analyses were performed using STATA SE 14.2.

## Results

The participants in the validation study were similar to those in the FRESH Austin study with respect to age, ethnicity and income, with no significant differences in these characteristics between study populations. Validation study participants were significantly more female and spoke different languages at home, especially a language other than English, Spanish, or English and Spanish equally (Table 2). Mode of data collection (in-person or via telephone) for the FFQ was not statistically different between FRESH Austin and validation study participants.

**Table 2:** Comparison of selected demographic characteristics of validation study and FRESH Austin participants

	Validation study, (*n* 54)	%	FRESH Austin, (*n* 400)	%	*P*
Sex					
Female	45	78·33	282	70·50	0·01
Age in years					
Mean	43·56		43·89		0·87
sd	1·89		0·68		
Ethnicity					
Hispanic	12	22·22	127	31·99	
Black	3	5·56	32	8·06	
White	29	53·70	203	51·13	
Other	10	18·52	35	8·82	0·09
Income					
Less than $25 001	12	23·53	89	23·30	
$25 001–$45 000	21	41·18	112	29·32	
$45 001–$65 000	8	15·69	70	18·32	
> $65 000	10	19·61	111	29·06	0·30
Language at home					
Only/mostly English	24	44·44	236	59·15	
Both English and Spanish	10	18·52	51	12·78	
Only/mostly Spanish	16	29·63	109	27·32	
Mostly other	4	7·41	3	0·75	<0·01
FFQ data collection mode					
In-person	48	88·89	311	77·75	
By telephone	6	11·11	89	22·25	0·06

FRESH Austin, Food Retail: Evaluating Strategies for a Healthy Austin.

A comparison of crude estimates of FFQ and 24hDR servings/d indicates that ‘Non-Citrus Fruits’ and ‘Other Vegetables’ have the highest values across both instruments, reflecting the inclusion of a variety of FV in each category. The FFQ and 24hDR produced similar estimates of average total servings/d across FV (6·68 and 6·40 servings/d, respectively,) as well as for individual FV categories. Further comparison of crude estimates via the paired *t*-test reveals that there were no significant mean differences between total fruit, total vegetables and total FV. In all, no FV categories had significant mean differences (Table 3.)

**Table 3:** Crude mean servings and sD for each food category (per day) by assessment method (FFQ and 24hDR), and paired *t*-test

Food category	FFQ (servings/d)		24hDR (servings/d)		Paired *t*-test
	Mean	sd	Mean	sd	*P*
Fruit					
Citrus	0·68	0·12	0·30	0·08	0·17
Non-citrus fruits	2·06	0·20	2·29	0·21	0·32
AVG total fruit	2·74	0·21	2·59	0·17	0·20
Vegetables					
Avocados	0·28	0·05	0·13	0·04	0·35
Dark green	0·41	0·09	0·61	0·10	0·28
Deep yellow	0·44	0·07	0·37	0·05	0·08
Tomatoes	0·46	0·07	0·54	0·07	0·88
White potatoes	0·25	0·06	0·30	0·05	0·12
Starchy	0·22	0·05	0·15	0·03	0·11
Other vegetables	1·88	0·21	1·71	0·14	0·49
AVG total vegetables	3·94	0·13	3·81	0·10	0·26
AVG total FV	6·68	0·14	6·40	0·13	0·51

24hDR, 24-h dietary recalls; FV, fruit and vegetables.

Across categories of FV, the FRESH Austin FFQ provided moderately correlated outcomes compared with the repeated 24hDR, with deattenuated correlations above 0·30 for all food groups, except potatoes (Table 4). High correlations were observed for ‘Non-Citrus Fruits’ and ‘Other Vegetables’, which were both above 0·50. All correlations were significant, except for ‘White Potatoes’ and ‘Avocados’, and 55 % of food categories had correlations above 0·40. The highest correlations were found for total fruit (0·69), total vegetables (0·79) and total FV (0·73) (Table 4).

**Table 4:** Spearman’s *ρ* or Pearson’s *r* mean absolute percentage error (MAPE), based on servings/d, *n* 56

Food category	Spearman *ρ*/Pearson’s *r*	*P*	Deattenuated correlation	*P*	MAPE (%)	sd
Citrus	0·48	<0·01	0·51	0·01	3·0	0·06
Non-citrus Fruits	0·57	<0·001	0·60	0·004	0·9	0·10
Total fruit	0·64	<0·001	0·69	<0·001	2·2	0·05
Avocados	0·38	0·38	0·40	0·43	3·5	0·05
Dark green	0·33	0·02	0·34	0·02	0·7	0·03
Deep yellow	0·51	<0·01	0·56	0·02	1·1	0·06
Tomatoes	0·47*	<0·01	0·47	0·03	0·9	0·05
White potatoes	0·01	0·93	0·12	0·61	2·6	0·10
Starchy	0·30	0·03	0·32	0·02	1·5	0·03
Other vegetables	0·59*	<0·001	0·59	<0·001	0·4	0·10
Total vegetables	0·74	<0·001	0·79	<0·001	1·7	0·06
Total FV	0·69	0·02	0·73	0·03	0·7	0·06

* Pearson’s *r*, variable normally distributed after log-transformation.

Because our interest is in a measure of error that does not penalise larger magnitude errors more than smaller magnitude errors, the MAPE was utilised to provide further insight. The MAPE relies on average values, meaning that large deviations are not as influential, allowing for frequently observed daily variances in consumption to be captured in the 24hDR and integrated more realistically in comparison to habitual dietary consumption patterns as recorded by the FFQ. MAPE values were small for all FV, suggesting the variance of the error estimates was also small. In addition, these data indicate that the errors are small in both the negative and the positive direction, which is important to the assessment of agreement between the two methods, where either a positive or a negative difference would be of interest. The larger values generated for ‘Avocados’ and ‘White Potatoes’ suggest that larger errors in assessment may make it more difficult to capture significant changes for these categories, while the small MAPE values for ‘Non-Citrus Fruits’ and ‘Other Vegetables’ suggest these categories reflect more accurate measures of FV consumption.

**Fig. 2:**
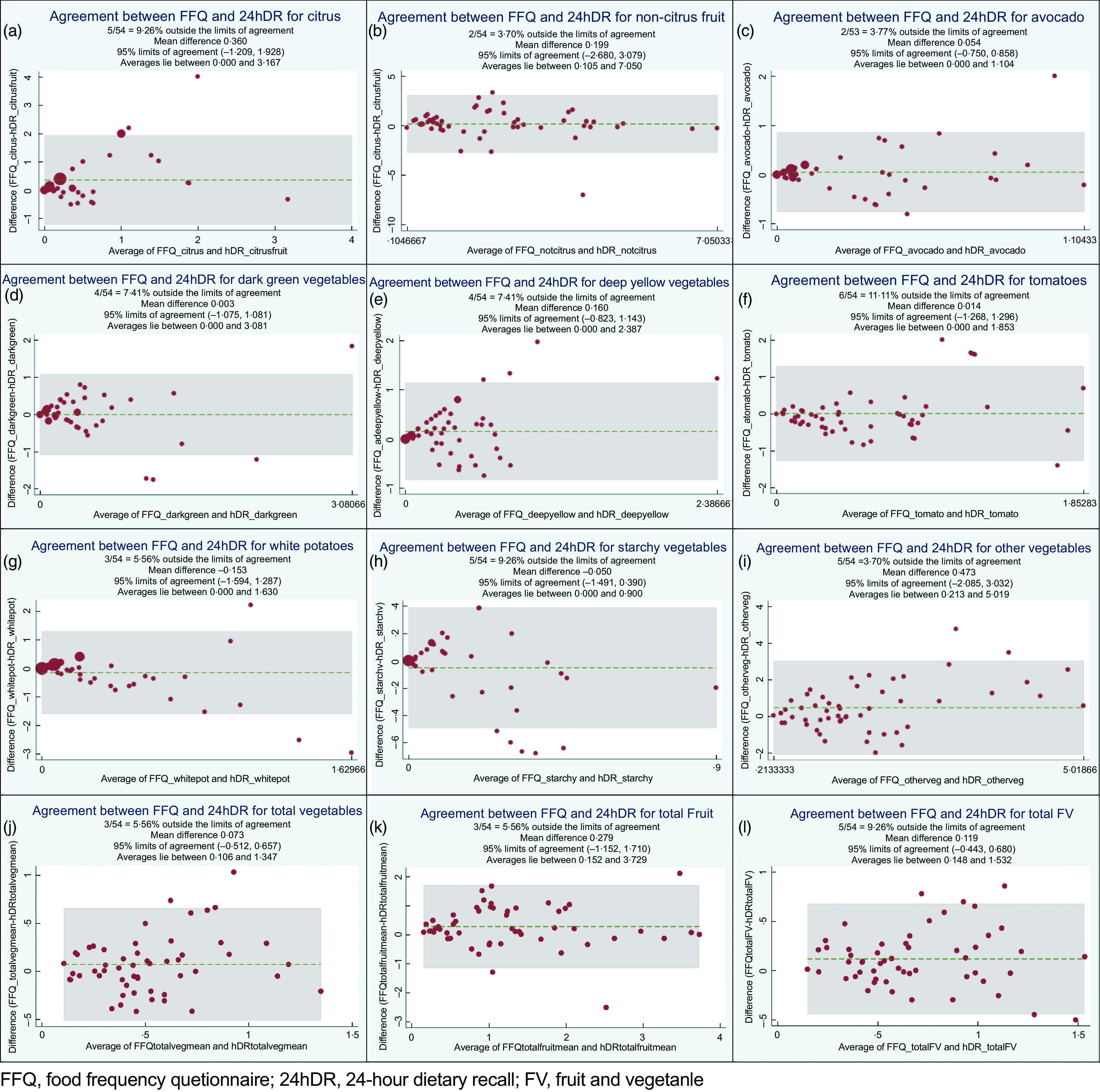
Bland–Altman plots comparing the average of daily serving differences between FFQ and 24-h dietary recall (24hDR) estimates for all FV categories

Finally, Bland–Altman plots (Fig. 2) provide greater detail for assessing the degree to which the two methods agree. Each plot shows the line of equality, or the line upon which all points would appear if the FFQ and the 24hDR produced the exact same measure^(30,31)^. Thus, these plots provide a visual method of assessing within-subject variance. For every food category, the plots show the grouping of data points for smaller values to be closer together, while outliers are generally found at larger values. Mean differences were less than 0·50 servings/d for all categories, including the ‘Non-Citrus’ and ‘Other Vegetables’ categories, which have larger crude values due to the inclusion of a greater variety of FV. Limits of agreement for each food category describe the range of agreement among the FFQ and 24hDR for 95 % of individuals assessed^(32)^.

## Discussion

The central question of the current study is whether the FRESH Austin FFQ provides a valid measure of FV consumption, compared with the 24hDR^(33)^. This is motivated by the desire to use the least burdensome dietary assessment method that is sufficiently accurate to detect differences in FV consumption. The 24hDR is often used as the reference method compared with the FFQ for several reasons: it has less reliance on long-term memory than the FFQ (requiring recall of only the previous day), utilises a trained interviewer to enhance details and accuracy and elicits a detailed record of consumption, including methods of preparation and details of brands and sources^(7,19,34)^. However, the strengths of the 24hDR also make it burdensome, requiring repetition and trained personnel to achieve validity in line with established protocols. A consistent agreement of the FRESH Austin FFQ and repeated 24hDR allows the use of the less burdensome option, in this case the FFQ, to be deemed reliable and effective at detecting important changes in consumption in the population of interest^(35,36)^.

In previous studies, the FFQ generally overestimates usual intake^(6–8,19,35,37)^ and yields correlations between 0·40 and 0·70 across food groups and nutrients, compared with the 24hDR^(38)^. In our study, we found correlations between 0·30 for ‘Starchy’ vegetables and 0·79 for ‘Total Vegetables’, indicating that the FRESH Austin FFQ provides moderately valid measures for FV surveyed, except for ‘Potatoes’, which had a very low correlation of 0·12. We suspect this low correlation is due to NDSR software separating fried from other potatoes and combining fried starchy vegetables with fried potatoes, while the FFQ did not differentiate.

Our study found correlations in line with similar research, such as Hebden *et al.*’s^(39)^ comparison of a tailored FFQ to repeated food records. In that study, fruit servings were correlated (*r* = 0·58) in line with our results (‘Citrus’ *r* = 0·51, ‘Non-Citrus’ *r* = 0·60), as were vegetable servings (*r* = 0·57) in comparison to our ‘Other Vegetables’ (*r* = 0·59)^(39)^. As found in other studies, the FFQ slightly overreported the consumption of FV compared with the criterion measure^(5,6,9,39,40)^. This may be attributed to social desirability bias, as participants may report habitual intake to resemble their own intended consumption or their perceptions of the interviewer’s expectations, rather than actual intake. In contrast, the 24hDR recall may reduce this bias by asking more immediate questions of recent intake, providing less opportunity to edit consumption to align with intentions. Further, as noted by Boucher *et al.*, higher numbers of recall days are associated with greater correlations with FFQ values, suggesting that for some food categories, more than two or three recalls are required to account for daily variance in consumption^(37)^. While the brevity of the FRESH Austin FFQ reduced survey burden, it also eliminated the ability to calculate total energy, since the entire diet was not evaluated. This limitation would be important for any investigations into associations with disease, since consumption cannot be scaled by total energy. However, the truncated FFQ may be appropriate for studies intended to capture changes in FV consumption, rather than deattenuated values^(41,42)^.

Agreement between the FFQ and 24hDR was reported via the paired *t*-test, which found that none of the mean differences in any FV category were significantly different from zero (i.e., all comparisons were nonsignificant). In addition, the small error estimates obtained via the MAPE suggest the two methods substantially agree.

The pattern observed via the Bland–Altman plots, showing closer agreement at smaller quantities and greater discrepancies at higher quantities, argues for the careful examination and possible exclusion of outliers in pre-/post-assessments. Similar results were reported in other studies^(43–45)^ where greater variation was found at higher levels of intake. These values may be ‘true’, in the sense that large quantities of a specific food were eaten, but ‘untrue’ as an indicator of habitual consumption. Because measures of agreement in the Bland–Altman plots are distributed both above and below the line of equality, no systematic bias is detected. However, the limits of agreement were wide (Fig. 1, panels (a)–(1)), which is similar to research reported in two studies by Conway *et al.* ^(16,17)^, Bautista *et al.* ^(46)^ and a comparable validation study among Lebanese children^(43)^, suggesting that the FFQ may lead to important under- or over-estimation of actual intake. This feature, as well as the truncated food list of the FFQ (only FV, rather than the whole diet), limits the use of this instrument in exploring associations with chronic disease, where precise and calibrated nutrient estimates are critical. However, because mean estimates of FV consumption exhibit no indication of systematic bias, this FFQ would be appropriate for valid comparisons of cohort designs^(46)^. This FFQ would be useful to provide estimates of changes in usual consumption over time, where the outcome of interest is not a measure of true intake, but rather an assessment of changes in consumption. The lower survey burden and moderate precision of the FFQ may be well-suited to community-level interventions that are intended to change consumption patterns.

Limitations of the current study include a lack of energy adjustment from total kcal, which was not possible because the entire diet was not assessed via the FFQ. This limits the ability to make conclusions regarding associations with disease, since the results cannot be scaled by total consumption. In addition, the FFQ food list and NDSR software were not aligned for ‘Potatoes’, with NDSR separating fried from other potatoes, whereas the FFQ did not. We suspect this is the cause of the low correlation in this category and may not indicate a true problem with the FFQ. More worrisome, however, is the low correlation for avocados, which can be an important part of the diet for Hispanic populations. Future research may need to explore better ways of capturing this category of consumption more accurately, perhaps including descriptions of how avocados may be consumed, such as in dips or sauces. Our study protocol may have introduced bias by setting up appointments for subsequent data collections, increasing the opportunity for social desirability bias to affect dietary behaviours in advance of our interviews. The current study’s strengths include using an FFQ food list that was carefully adapted to the population of interest, fully bilingual materials and research staff, and multiple methods of analyses (*t*-tests, correlation, MAPE and Bland–Altman graphs) to explore the validity of the FFQ.

## Conclusion

Every FV group assessed by the FRESH Austin FFQ showed acceptable levels of association between FFQ and 24hDR, with the exception of potatoes and avocadoes, suggesting that this tailored FFQ is able to capture usual consumption with sufficient accuracy to enable valid assessment of changes in FV intake. The FFQ minimises respondent burden, which is an especially important condition for retention in cohort studies and helps ensure sufficient sample sizes and power to detect changes in outcomes. In addition, the FRESH Austin FFQ’s focus on whole foods is aligned with evaluation of community-based interventions, such as Austin’s FFL programme, aimed at improving access in high-need communities^(47–49)^. As in other community-level programming, the outcomes of interest are changes in patterns of consumption, specifically increases in FV intake. This FFQ aligns evaluation with implementation, providing a measure of change that is important for programme evaluation, as well as for assessment of an important determinant of desired health outcomes.
